# Identification of human DP8α regulatory T cell sub-populations reactive to health-associated anti-inflammatory gut commensals

**DOI:** 10.1080/19490976.2026.2690686

**Published:** 2026-06-28

**Authors:** Margaux de Seilhac, Margaux Verdon, Lucas Goisnard, Aurore Duquenoy, Nathalie Corvaia, Emilie Plantamura, Nicolas Lapaque, Fabienne Beguet-Crespel, Bastien Laperrousaz, Joel Doré, Frédéric Altare, Emmanuelle Godefroy

**Affiliations:** a Nantes Université, Univ Angers, INSERM, CNRS, Immunology and New Concepts in ImmunoTherapy, INCIT, UMR 1302, Nantes, France; b LabEx IGO/IMMUNE, Nantes Université, Nantes, France; c MaaT Pharma, Lyon, France; d Micalis Institute, INRAE, AgroParisTech, University Paris-Saclay, Jouy-en-Josas, France

**Keywords:** Gut microbiota, regulatory T cells, human gut microbiota-specific Tregs, dendritic cells, immune regulation, *Faecalibacterium duncaniae*, *Akkermansia muciniphila*, *Blautia*, *Roseburia intestinalis*

## Abstract

While the role of gut microbiota in health has been demonstrated, deciphering the involvement of individual bacterial species in diseases with unmet clinical needs represents a major research interest. Over the last decade, DP8α regulatory T cells (Tregs) have been identified to respond to *Faecalibacterium duncaniae* and protect against inflammatory bowel diseases (IBD) and acute graft-*versus*-host disease (aGvHD). To better understand, on the one hand, physiological DP8α Treg activation processes, and, on the other hand, the underlying mechanisms used by anti-inflammatory bacterial species to protect the host, we investigated whether additional key species could be recognized by DP8α Tregs. Here we showed that *Blautia obeum*, *Roseburia intestinalis,* and *Akkermansia muciniphila* indeed represent sources of antigens for DP8α Treg activation and priming, likely through their unique ability to induce tolerogenic antigen presenting cells. This study provides considerable mechanistic insight into the gut microbiota/regulatory T cell interplay and further establishes the role of DP8α Tregs, and their emerging dedicated ability to respond to gut microbiota, in local and systemic homeostasis.

## Graphical abstract

**Figure:**
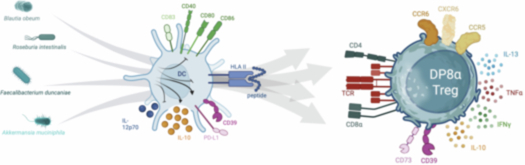

## Introduction

As gut microbiota diversity has evidently been linked to health and susceptibility to numerous diseases,^1^ ^,^ ^2^ understanding the role of individual bacterial species within this complex ecosystem represents a significant challenge. Several members of the class Clostridia exert anti-inflammatory functions and can induce regulatory T cells (Tregs). For instance, a panel of 17 Clostridia was demonstrated to induce CD4^+^/FoxP3^+^ Tregs and to alleviate colitis in mice.^3-5^ In humans, another class Clostridia member, *Faecalibacterium duncaniae* A2-165 (*F. duncaniae* A2-165, previously known as *Faecalibacterium prausnitzii* A2-165), triggers IL-10 production^6^ ^,^ ^7^ and promotes antigen presenting cells (APCs) with tolerogenic properties through a variety of pathways.^8^ Our team has identified, in 2014, a human Treg subset, named DP8α Tregs, with TCR specificities for *F. duncaniae* A2-165,^9-11^ likely through the anti-inflammatory and tolerogenic properties mentioned above.

Unlike mouse microbiota-reactive Foxp3^+^ Tregs, the heterogenous subset of human Foxp3^+^ Tregs, does not seem particularly prone to respond to gut microbiota in a TCR-dependent manner. While peripherally induced Foxp3^+^ Tregs (pTregs) are involved in gut homeostasis, data regarding their antigenic specificities remain elusive.^12^ Moreover, their clinical use remains challenging, mainly due to their limited proliferative ability, instability and lack of specific human markers.^13^ ^,^ ^14^ Other Foxp3^-^ Treg subsets, including Tr1-like Tregs, which can also differentiate from peripheral naive CD4^+^ T cells,^15^ exert suppressive functions, although through contact-independent mechanisms largely relying on cytokines like IL-10. The clinical potential and physiological relevance of Tr1-like Tregs should unravel as enhanced methods to produce pure and stable Tr1 cells arise, recognized antigens characterized and specific reliable markers identified.^16^

The suppressive *F. duncaniae*-reactive DP8α Treg subset, strongly enriched in the CCR6^+^/CXCR6^+^ fraction of CD4^+^/CD8α^LOW^ DP8α T cells,^11^ was found to be drastically altered in two main pathologies, namely inflammatory bowel diseases (IBD) and acute graft-*versus*-host disease (aGvHD), initially suggesting that CD3^+^/CD4^+^/CD8α^LOW^/CCR6^+^/CXCR6^+^ DP8α T cells, herein named DP8α Tregs, could be involved in the protection against both conditions.^9-11^ ^,^ ^17^ Interestingly, the abundance of *F. duncaniae* has consistently been reported to be decreased within the gut microbiota of these patients.^16^ ^,^ ^18-25^ These data then prompted preclinical studies, which supported the ability of DP8α Tregs to alleviate DSS-induced colitis^26^ and drastically protected against aGvHD^17^ in humanized mouse models. *F. duncaniae*-reactive DP8α Tregs therefore appeared to be a key player in gut homeostasis.

Several diseases, including IBD and aGvHD, have been associated with decreased abundances of additional species belonging to other genera, such as *Blautia* and *Roseburia*, which, like *F. duncaniae*, belong to the class Clostridia and Bacillota [Firmicutes] phylum,^27-30^ as well as the *Akkermansia* genus belonging to the Verrucomicrobiota phylum.^31-33^ Furthermore, *Akkermansia muciniphila* (*A. muciniphila*) shares with *F. duncaniae* the ability to promote anti-tumor immunity and significantly improve the clinical response to immune checkpoint blockade in certain cancers.^6^ ^,^ ^34-37^ These data therefore provide a significant rationale to investigate the interplay between such bacteria and DP8α Tregs.

We selected 3 key anti-inflammatory species from these genera, *Blautia obeum* (*B. obeum*), *Roseburia intestinalis* (*R. intestinalis*) and *A. muciniphila*, which have been associated with a decreased risk of IBD and aGvHD among other ailments,^27-30^ ^,^ ^32^ ^,^ ^38-40^ and explored, here, their potential ability to be recognized and to stimulate DP8α Tregs. Indeed, these species not only embody promising candidates to target in various inflammatory and metabolic diseases, whose underlying mechanism(s) still need to be uncovered, but also could start unravelling the ontogeny and the physiological relevance of this recently identified DP8α Treg subset.

## Materials and methods

### Isolation and generation of mo-DCs from healthy volunteers' PBMCs

Blood samples were collected into platelet-leukocyte concentrates from healthy volunteers at the Nantes Blood Center (EFS, CPDL-PLER-2021-109). PBMCs were isolated by Ficoll gradient centrifugation (lymphocyte separation medium, EurobioScientific). Monocytes were purified from PBMCs using anti-CD14 microbeads (Miltenyi Biotec) and were differentiated into monocyte-derived dendritic cells (mo-DCs) by a 5- to 6-d culture with 1000 IU/mL rhGM-CSF (Granulocyte-Macrophage Colony-Stimulating Factor) and 300 IU/mL rhIL-4 (CellGenix). Non-adherent immature DCs were harvested at day 5–6 and cultured with or without inactivated bacterial strains (ratio 1 DC:25 bacteria) and/or with or without 10 μM R848 (TLR7/8 agonist). When the TLR agonist was used, DCs were incubated with bacterial strains overnight, followed by the addition of R848 for 48 h. In the absence of the agonist, DCs were exposed to bacterial strains for the entire duration of the co-culture without any additional agent.

### Bacteria

Bacterial strains from *F. duncaniae*, *A. muciniphila, R. intestinalis*, *B. obeum,* and *Blautia luti (B. luti)* species were obtained either from the “Functionality of the Intestinal Ecosystem” team at the Micalis Institute (INRAE, Jouy-en-Josas, France) or isolated from fecal samples of healthy donors provided by MaaT Pharma (Lyon, France) as part of a collaborative project, and processed by the Bioaster Institute (Paris, France) (Table 1). Bacteria were heat-inactivated using three temperature cycles consisting of 5 min at 4 °C followed by 5 min at 95 °C.

### Flow cytometry

For surface staining, cells were washed and stained for 45 min at 4 °C in Phosphate Buffered Saline (PBS) (PanReac AppliChem) containing 0.1% Bovine Serum Albumin (BSA) (Sigma Aldrich) with antibodies targeting the following markers: CD3 (clone UCHT1, Becton-Dickinson), CD4 (clone 13B8.2, Beckman-Coulter), CD8α (clone RPA-T8, Becton-Dickinson), CCR5 (clone 2D7, BD Biosciences), CCR6 (clone G034E3, BioLegend), CXCR6 (clone K041E5, BioLegend), CD39 (BD Biosciences, TU66 clone), CD73 (clone AD2, BioLegend), CD25 (BD Biosciences, M-A251 clone), CD127 (clone HIL-7R-M21, BD Biosciences), CD14 (clone M5E2, BioLegend), CD11c (clone Bu15, BioLegend), CD83 (clone HB15e, BioLegend), CD80 (clone 2D10, BioLegend), CD86 (clone 2331, BD Biosciences), CD40 (clone 5C3, BioLegend), PD-L1 (clone MIH1, BD Biosciences), and class II HLA (DP, DQ, DR) (clone WR18, Invitrogen).

For intracellular staining, cells were fixed in 4% paraformaldehyde (Electron Microscopy Sciences) for 10 min at room temperature (RT), washed and stained at RT for 40 min in PBS/0.1% BSA/0.1% saponin with antibodies targeting the following cytokines: TNFα (clone Mab11, Invitrogen), IFNγ (clone B27, BD Bioscience), IL-13 (clone JES10-5A2, BioLegend), IL-4 (clone MP4-25D2, BD Bioscience), IL-17 (clone BL168, BioLegend), and IL-21 (clone 3A3-N2, Invitrogen).

For transcription factors, cells were fixed and permeabilized using the FoxP3/Transcription Factor Buffer Set (eBioscience) with an antibody against FoxP3 (clone PCH101, Invitrogen).

Fluorescence was measured with a BD LSRFortessa™ flow cytometer and analyzed using the FlowJo software (version 10.9.0).

### ELISA

The levels of IL-10 and IL-12p70 were quantified in cell culture supernatants by specific sandwich enzyme-linked immunosorbent assays (ELISA, BioLegend and R&D systems, respectively), according to the manufacturer's guidelines.

### CD4^+^ T cell priming

Naive CD4^+^ T cells were isolated from PBMCs using a selection kit (eBioscience), then stained with 1 μM Violet Proliferation Dye 450 (VPD, BD Bioscience) and co-cultured with allogeneic DCs (ratio 5:1) previously exposed or not to bacteria overnight and matured with R848 for 48 h. After 11 d, CD4^+^ T cells were re-stimulated with anti-CD3/CD28-coated beads (Gibco). IL-10 secretion was assessed on VPD^LOW^ primed T cells using IL-10 secretion assay—detection kit in accordance with the manufacturer's instructions (Miltenyi).

### Proliferation assay

CD14^+^ monocytes, and CD4^+^ T cells (comprising double-positive DP8α Tregs) were sorted from healthy donors' PBMCs using magnetic beads (Miltenyi Biotec). CD4^+^ T cells were stained with 1 μM VPD and were then co-cultured with CD14^+^ autologous monocytes (ratio 1:1) previously loaded overnight with inactivated bacteria (ratio 1 monocyte:5 bacteria) in the presence of low dose rhIL-2 (20 IU/mL; Proleukine, Novartis). Five days later, T cell proliferation was measured through VDP dilution assessment by flow cytometry.

### Production of human Treg clones

To isolate bacteria-reactive DP8α Treg clones from blood, purified VPD-stained CD4^+^ T cells, comprising DP8α Tregs, were co-cultured with purified autologous CD14^+^ monocytes loaded overnight with *F. duncaniae* A2-165, *A. muciniphila* DSM 22959, *B. obeum* DSM 25238 or *R. intestinalis* DSM 14610 (1:5 ratio). Five days later, VPD^LOW^ CD3^+^/CD4^+^/CD8α^LOW^ cell clones were produced using the FACS Aria III cell sorter. Clones were then amplified on feeder cells, as previously described.^11^ Briefly, feeder cells were made from irradiated (40 grays) allogeneic PBMCs and the irradiated (70 grays) immortalized (with EBV) LAZ cell line, at a 10:1 ratio. Reactive VPD^LOW^ single DP8α T cells (= clones) were distributed on these feeder cells (10^5^ feeders per well, in U-bottom 96-well plates), using the Aria III Cell Sorter. Cells were then cultured in complete RPMI-1640 medium containing 8% heat-inactivated human serum (Pan Biotech, Seraclot, Type AB, Male), 2 μM L-glutamine (Sigma), 100 UI/mL streptomycin/100 μg/mL penicillin (Sigma), and 200 IU/mL rhIL-2 (AstraZeneca), in the presence of 1 μM phytohemagglutinin (Sigma). Cultures were checked daily and divided in fresh medium when needed. After a 4-week long amplification on feeders, clonality was confirmed through TCR Vβ staining (IOTest® Beta Mark TCR V beta Repertoire kit, Beckman Coulter), showing that populations were >99% clonal, i.e., expressing a single TCR Vβ chain (Figure S1). Resting clones were then screened for their response to autologous monocytes presenting bacteria, and all other presented experiments.

### T cell clone cytokine production

T cell clones were stimulated by autologous monocytes (1 lymphocyte:1 monocyte ratio) loaded overnight or not with bacteria (5 bacteria:1 monocyte ratio). For TNFα, IFNγ, IL-13, IL-4, IL-17, and IL-21 detection, T cell clones were stimulated for 6 h in the presence of 10 μg/mL brefeldin A before intracellular staining of cytokines. For IL-10 detection, clones were stimulated for 48 h before IL-10 measurement by enzyme-linked immunosorbent assay (ELISA). For blocking experiments, we used anti-HLA class II ascites-derived polyclonal antibodies (“206”) produced in our laboratory.

### Inhibition of T cell proliferation

Freshly sorted CD4^+^ T cells (Miltenyi) were stained with 1 μM VPD before being co-cultured with Treg clones in 20 UI/ml rhIL-2, with or without CD3/CD28 microbeads (Miltenyi). VPD dilution was assessed 5 d later to evaluate proliferation.

### Statistics

Statistical significances were conducted with GraphPad Prism (software 10.1.2(324)). Depending on comparisons, Mann–Whitney U tests, Wilcoxon tests or Kruskal–Wallis tests with Dunn's post hoc analysis were applied, as indicated in the Figure legends. Results were considered statistically significant when *p*-values < 0.05.

## Results

### DP8α T cells can respond to *Akkermansia muciniphila*

DP8α Tregs have originally been identified to respond to *F. duncaniae* A2-165 in a TCR-dependent manner.^9-11^ To start investigating whether these DP8α Tregs could recognize additional gut microbiota commensals involved in key physiological processes promoting health, we first tested by flow cytometry *ex vivo* polyclonal CD4^+^ T cells, encompassing DP8α cells, derived from healthy donors (HDs), for their proliferative responses to *A. muciniphila* DSM 22959 and, alongside, to *F. duncaniae* A2-165 as a reference.

**Figure 1.**
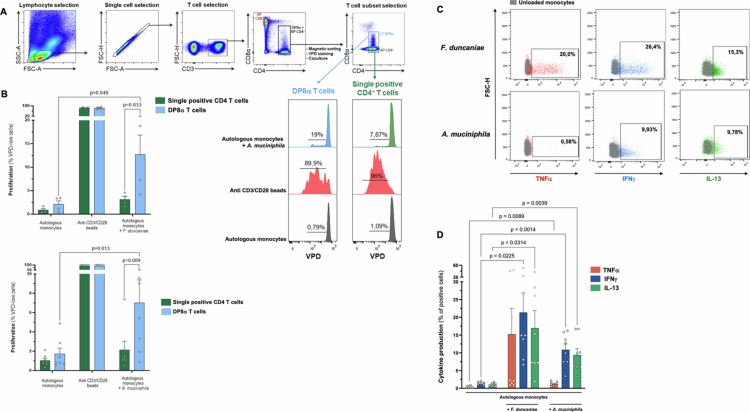
DP8α T cells can respond to *Akkermansia muciniphila.* (A,B). Gating strategy for PBMCs from healthy donors to gate and to purify total CD4⁺ T cells (encompassing DP8α Tregs), which were labeled with VPD and co-cultured with magnetically sorted autologous monocytes (1:1), preloaded overnight or not with inactivated bacteria from *A. muciniphila* DSM 22959 or *F. duncaniae* A2-165 (monocyte:bacteria ratio 1:5), in the presence of low-dose (20 IU/ml) rhIL-2 (A). Reactive T cells (within DP8α or SP CD4^+^ T cells) were detected through VPD dilution shown in histogram plots (A) and represented in quantitative graphs (B). It is worth noting that, DP8α Tregs only represent CD4^HIGH^ T cells co-expressing low levels of CD8α. Indeed, only double-positive cells with low levels of CD8α contained the microbiota-reactive Treg subset.^9-11^ Double-positive cells with high levels of CD8α are not comprised in the DP8α gate as these cells have a different phenotype, e.g., they do express CD8β, unlike DP8α (hence their name), are not regulatory, produce inflammatory cytokines, are HLA class I-restricted (DP8α Tregs are HLA class II-restricted) and exhibit reactivity to tumor antigens in the context of colorectal cancer.^9^ ^,^ ^10^ ^,^ ^50^ Five days later, T cell proliferation was assessed through VPD dilution analysis. The gating strategy used to study the DP8α or single-positive CD4^+^ T cell proliferation is shown, as well as an example of a representative donor (A). Proliferation in response to *A. muciniphila* DSM 22959 or *F. duncaniae* A2-165 is represented for all donors in mean percentages of VPD^LOW^ cells ± SEM (B). (C and D). Eight *F. duncaniae*-reactive DP8α Treg clones obtained from the blood of healthy donors were screened for their production of TNF-α, IFN-γ, and IL-13 to autologous monocytes loaded with *F. duncaniae* A2-165 or *A. muciniphila* DSM 22959. An example of cytokine production is shown for one *F. duncaniae*-reactive DP8α Treg clone (C) and data for all 8 *F. duncaniae*-reactive DP8α Treg clones are represented in mean percentages of positive cells for each cytokine ± SEM (D). Statistical significances were assessed using two-way ANOVA analyses (B and D). *p*-values < 0.05 were considered significant.

Briefly, sorted total CD4^+^ T cells were stained with the Violet Proliferation Dye (VPD) and co-cultured with CD14^+^-sorted autologous monocytes, as APCs, previously loaded with either *A. muciniphila* DSM 22959 or *F. duncaniae* A2-165. Five days later, proliferated VPD^LOW^ DP8α cells were detected in response to each bacterial species (Figure 1). Five HDs out of the 7 tested presented fractions of DP8α cells responding to *A. muciniphila* DSM 22959 (mean = 9.3%, ranging from 3.3% to 19%), as compared to autologous monocytes not exposed to any bacteria (mean = 1.7% ± 0.61, *p* = 0.013, Figure 1A and B). Of note, little to no *A. muciniphila* DSM 22959 responses were detected in single-positive (SP) CD4^+^ T cells (Figure 1A and B). These responses to *A. muciniphila* DSM 22959 were comparable to the ones observed against *F. duncaniae* A2-165, i.e., preferably found in DP8α T cells, but not in SP CD4^+^ T cells (Figure 1B), as our team has previously shown.^9^ ^,^ ^11^ These data strongly support the ability of DP8α T cells, initially identified to respond to *F. duncaniae* A2-165,^9^ to also recognize and respond to another gut microbiota commensal, namely *A. muciniphila* DSM 22959.

To unambiguously demonstrate the actual existence of *A. muciniphila*-reactive DP8α T cells and explore whether *F. duncaniae*-reactive DP8α Tregs cross-reacted with *A. muciniphila* or whether distinct populations of DP8α T cells harbored different bacterial specificities, VPD^LOW^ cells from gated DP8α T cells proliferating against autologous monocytes loaded with *F. duncaniae* A2-165 were single-cell sorted using the Aria III cytometer. Eight *F. duncaniae*-reactive DP8α T cell clones, from 2 donors, were obtained and successfully amplified on feeder cells, as previously described^11^ and detailed in the Methods' Section (Figure S1). The 8 clones obtained produced various levels of TNFα, IFNγ and IL-13 and responded to *F. duncaniae* A2-165 by producing significant levels of at least 1 cytokine (Figure 1C and D), in accordance with what was previously described.^11^ Using those three cytokines, we screened the 8 DP8α T cell clones for their potential cross-reaction with *A. muciniphila*. Interestingly, most clones also responded to *A. muciniphila* DSM 22959 (Figure 1C and D), mainly by producing IFNγ and/or IL-13, suggesting that both bacteria could share T cell antigenic epitope(s).

### Characterization of *A. muciniphila*-reactive DP8α T cell responses

To study global and unbiased DP8α T cell responses directed against *A. muciniphila*, sorted VPD-stained total CD4^+^ T cells from 2 HDs were stimulated *ex vivo* by autologous monocytes loaded with *A. muciniphila* DSM 22959. Five days later, VPD^LOW^ DP8α T cell clones were produced (Figure S1). Eight clones were generated, representing valuable tools to characterize these *A. muciniphila*-reactive DP8α T cell responses. In parallel, the 8 *F. duncaniae*-reactive DP8α T cell clones were used as a reference, to compare clones of both specificities.

**Figure 2.**
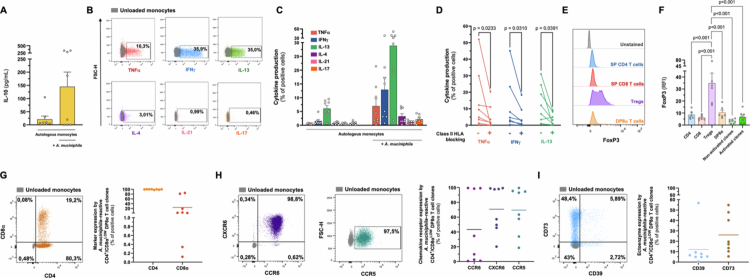
*A. muciniphila*–reactive DP8α T cell clone cytokine production and phenotype. Eight *A. muciniphila*-reactive DP8α T cell clones were obtained from healthy donors' PBMCs. Briefly, purified VPD-stained CD4^+^ T cells, comprising DP8α T cells, were co-cultured with purified autologous CD14^+^ monocytes loaded overnight with *A. muciniphila* DSM 22959. Five days later, VPD^LOW^ CD3^+^/CD4^+^/CD8α^LOW^ cell clones were produced from 2 HDs using the FACS Aria III cell sorter. (A–D). The eight *A. muciniphila*-reactive DP8α T cell clones were screened for their production of IL-10 by ELISA as well as TNF-α, IFN-γ, IL-13, IL-4, IL-17, and IL-21 by flow cytometry, in response to autologous monocytes loaded with *A. muciniphila* DSM 22959. IL-10 production of all 8 *A. muciniphila*-reactive DP8α T cell clones is represented in pg/mL ± SEM (A). An example of TNF-α, IFN-γ, IL-13, IL-4, IL-17, and IL-21 production of one representative *A. muciniphila*-reactive DP8α T cell clone is shown (B) and data for all *A. muciniphila*-reactive DP8α T cell clones are represented in percentages of positive cells for each of these 6 cytokines ± SEM (C). The production of TNF, IFN-γ, and IL-13 in the presence of the 8 *A. muciniphila*-reactive DP8α T cell clones has been screened in the presence of a class II HLA blocking antibody (D). (E and F). The expression of the FoxP3 transcription factor has been assessed on all *A. muciniphila*-reactive DP8α T cell clones and has been compared with the expression on polyclonal single positive CD4^+^ or CD8^+^ T cells, polyclonal DP8α T cells or CD4^+^/CD25^HIGH^/CD127^LOW^ polyclonal Tregs from 6 healthy donors. An example is shown for one donor (E) and data for the 8 *A. muciniphila*-reactive DP8α T cell clones and the 6 healthy donors tested are represented as mean relative fluorescence intensity (RFI) ± SEM (F). (G–I). The expression of CD4 and CD8α (G), CCR6, CXCR6, and CCR5 (H), as well as CD39 and CD73 (I) were assessed by flow cytometry. An example of marker expression is shown for one representative *A. muciniphila*-reactive DP8α T cell clone and data for all 8 *A. muciniphila*-reactive DP8α T cell clones are represented as percentages of expression. Statistical significances were assessed using Mann–Whitney U tests (A,C,D) or Kruskal–Wallis tests with Dunn's post hoc analyses (F). *p*-values < 0.05 were considered significant.

In response to *A. muciniphila* DSM 22959, 4 out of 8 DP8α T cell clones produced significant amounts of IL-10 (mean = 145 pg/ml ± 55.4) (Figure 2A) and all clones produced TNFα, IFNγ, and/or IL-13 (mean > 6%) (Figure 2B and C). Low to no IL-4, IL-21, and IL-17A (mean < 3%) were detected (Figure 2B and C). *F. duncaniae*-reactive DP8α T cell clones produced the same cytokines at comparable levels in response to *F. duncaniae* A2-165 (Figure S2A and B) than clones produced against *A. muciniphila*. To assess whether these DP8α T cell clones responded to *A. muciniphila* DSM 22959 in a TCR-dependent manner, as previously demonstrated for *F. duncaniae*-reactive DP8α T cells,^9^ their responses were tested in the presence of HLA-II blocking antibody. Their TNFα, IFNγ, and IL-13 production was significantly inhibited by the antibody (Figure 2D), strongly suggesting that the observed responses were mediated by TCRs recognizing *A. muciniphila*-derived epitopes presented by class II HLA molecules.

Next, we phenotypically characterized those *A. muciniphila*-reactive DP8α clones, based on key markers previously described to be expressed, or not, by *F. duncaniae*-reactive DP8α Tregs. Indeed, *F. duncaniae*-reactive DP8α Tregs have been found to be enriched in the CD3^+^/CD4^+^/CD8α^LOW^/CCR6^+^/CXCR6^+^ T cell fraction,^11^ to preferentially overexpress the anti-inflammatory-associated CCR5 receptor among total T cells in both blood and gut^41^ and to exert their suppressive function in a CD39/CD73-dependent manner.^11^ ^,^ ^17^ The expression patterns of these particular markers were therefore assessed. Similar to *F. duncaniae*-reactive DP8α Tregs, *A. muciniphila*-reactive DP8α T cell clones do not express FoxP3 (Figures 2E, F and S2C), expressed both CD4 and low levels (in terms of mean fluorescence intensity) of CD8α (Figures 2G and S2D) and displayed similar expression patterns of the chemokine receptors CXCR6 and CCR5, as well as similar expression patterns of the ectoenzymes mediating the purinergic pathway CD39 and CD73 (Figures 2 and S2E, F), as previously reported with *F. duncaniae*-reactive DP8α Tregs.^11^ ^,^ ^41^ Of note, *A. muciniphila*-reactive DP8α T cells displayed lower levels of CCR6 (mean = 43%, ranging from 1% to 99%) than *F. duncaniae*-reactive DP8α Tregs (mean = 75%, ranging from 1% to 100%). Heterogeneity of CCR6, CXCR6, and CCR5 expression observed between *A. muciniphila*-reactive DP8α T cell clones did not reflect any variation in their suppressive activity, suggesting these receptors do not directly impact the regulatory function of these cells.

**Figure 3.**
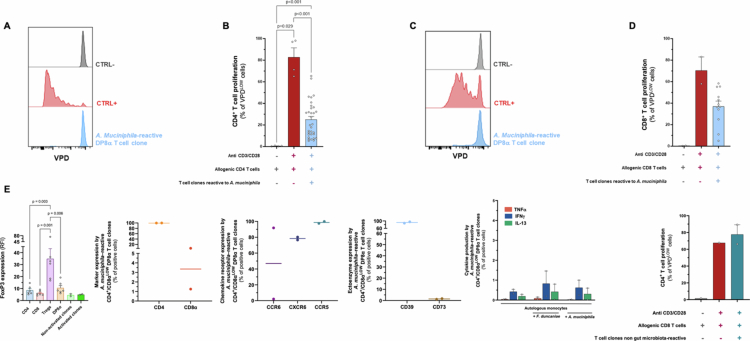
*A. muciniphila*–reactive DP8α T cell clones' immunoregulatory functions. (A–D). Allogeneic CD3/CD28-stimulated VPD-stained CD4^+^ or CD8^+^ T cells were co-cultured for 5 d with *A. muciniphila*-reactive DP8α T cell clones (ratio 1:1). Proliferation was evaluated by VPD dilution. An example is shown for CD4^+^ (A) and CD8^+^ T cells (C). Data for the 8 *A. muciniphila*-reactive DP8α T cell clones are represented for CD4^+^ (B) and CD8^+^ T cells (D) as percentages of VPD^LOW^ cells ± SEM. Statistical significances were assessed using Wilcoxon U tests (B and D). *p*-values < 0.05 were considered significant. (E). As a negative control, 2 FoxP3-negative CD4^+^ T cell clones, not selected for their commensal specificities, expressing low to no CD8α and CD73, and not producing cytokines (TNFα, IFNγ, IL-13) in response to autologous monocytes loaded with *F. duncaniae* or *A. muciniphila*, were included. Statistical significances were assessed using Wilcoxon U tests (B and D) or Kruskal–Wallis tests with Dunn's post hoc analyses (E). *p*-values < 0.05 were considered significant.

As *A. muciniphila*-reactive DP8α T cells closely resembled *F. duncaniae*-reactive DP8α Tregs in terms of cytokine production and phenotype, we next asked whether *A. muciniphila*-reactive DP8α T cells were suppressive Tregs. To do so, allogeneic VPD-stained CD4^+^ T cells from 4 distinct HDs were co-cultured for 5 d either alone or with each *A. muciniphila*-reactive DP8α T cell clones, in the presence of CD3/CD28 activation beads. Their proliferation was measured through VPD-dilution. Activated CD4^+^ T cells cultured alone displayed a mean proliferation of 82.9%, whereas co-cultures with *A. muciniphila*-reactive clones significantly reduced their proliferation to 24.9%, indicating that all clones inhibited CD4^+^ T cell proliferation in at least 3 out of 4 donors tested (Figure 3A and B), with a mean inhibition of 70% (*p* < 0.001) (Figure 3B). Similarly, *A. muciniphila*-reactive DP8α T cell clones also inhibited the proliferation of allogeneic CD8^+^ T cells from 2 HDs to varying degrees (mean inhibition = 52%) (Figure 3C and D). As controls, 2 FoxP3-negative CD4^+^ T cell clones, not selected for their commensal specificities, expressing low to no CD8α and CD73, and did not produce cytokines (TNFα, IFNγ, IL-13) in response to *F. duncaniae* nor *A. muciniphila*, were included. These clones did not inhibit allogeneic CD4^+^ T cell proliferation (Figure 3E), further connecting DP8α Treg commensal reactivity and suppressive activity. Therefore, *A. muciniphila*-reactive DP8α T cells suppress both CD4^+^ and CD8^+^ T cell proliferation, thereby exhibiting regulatory functions.

**Figure 4.**
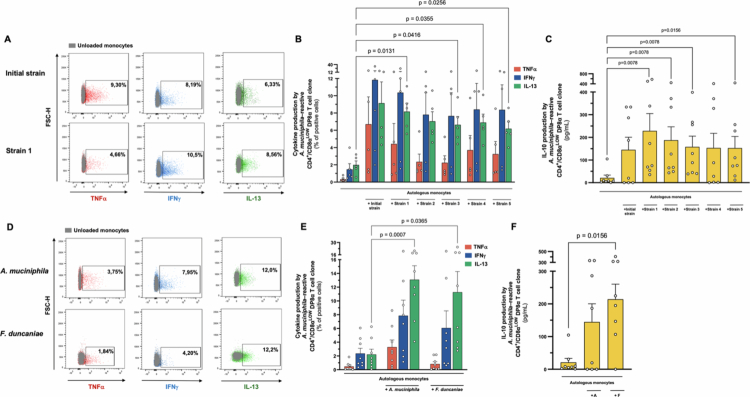
Human *A. muciniphila*–reactive DP8α Tregs exhibit cross-reactivity with distinct *A. muciniphila* strains and *F. duncaniae*. (A–C). *A. muciniphila*-reactive DP8α T cell clones were screened for their production of TNF-α, IFN-γ, IL-13, and IL-10 in response to autologous monocytes separately loaded ON with 6 *A. muciniphila* strains isolated from human feces: DSM 22959 (“initial strain”) as well as FAM 49 (strain 1), FAM 52 (strain 2), FAM 66 (strain 3), FAM 272 (strain 4), and FAM 278 (strain 5) strains. Representative examples of TNF-α, IFN-γ, and IL-13 production by one *A. muciniphila*-reactive DP8α T cell clone in response to the initial strain and strain 1 are shown (A) and data for all *A. muciniphila*-reactive DP8α T cell clones are represented in percentages of positive cells for each of these 3 cytokines ± SEM (B). The IL-10 production of all *A. muciniphila*-reactive DP8α T cell clones is represented in pg/mL ± SEM (background IL-10 production by bacteria-loaded monocytes cultured without T cell clones has been subtracted) (C). (D–E). The eight *A. muciniphila*-reactive DP8α T cell clones were screened for their production of TNF-α, IFN-γ, IL-13, and IL-10 in response to autologous monocytes loaded ON with *F. duncaniae* A2-165. Representative examples of TNF-α, IFN-γ, and IL-13 production by one *A. muciniphila*-reactive DP8α T cell clone in response to *A. muciniphila* and *F. duncaniae* are shown (D) and data for all *A. muciniphila*-reactive DP8α T cell clones are represented in percentages of positive cells for each of these 3 cytokines ± SEM (E). IL-10 production for all *A. muciniphila*-reactive DP8α T cell clones is represented in pg/mL ± SEM (background IL-10 production by bacteria-loaded monocytes cultured without T cell clones has been subtracted) (F). Statistical significances were assessed using two-way ANOVA analyses (B and E) or Wilcoxon U tests (C and F). *p*-values < 0.05 were considered significant.

To assess whether their reactivity extended beyond a single strain, *A. muciniphila*-reactive DP8α Treg clones were tested for their ability to respond to autologous monocytes loaded with 5 additional different *A. muciniphila* strains, isolated from the stools of 5 distinct HDs provided by MaaT Pharma and processed by the Bioaster Institute (Paris, France). All strains were recognized and induced DP8α Treg clones to produce TNFα, IFNγ, IL-13, and/or IL-10 (Figure 4A–C), suggesting that most recognized antigens are shared among *A. muciniphila* strains.

As DP8α Tregs selected for their response to *F. duncaniae* A2-165 also recognized *A. muciniphila* DSM 22959 (Figure 1C and D), we next asked whether DP8α Tregs generated against *A. muciniphila* DSM 22959 could reciprocally recognize *F. duncaniae*. All 8 DP8α Treg clones also responded to *F. duncaniae* A2-165 (Figure 4D–F). Of note, IL-10 production by these clones in response to *F. duncaniae* A2-165 (mean = 214 pg/mL ± 46.4) tended to be higher than in response to *A. muciniphila* DSM 22959 (mean = 145 pg/mL ± 55.4) (Figure 4F).

### DP8α T cell responses to *Blautia obeum* and *Roseburia intestinalis*

**Figure 5.**
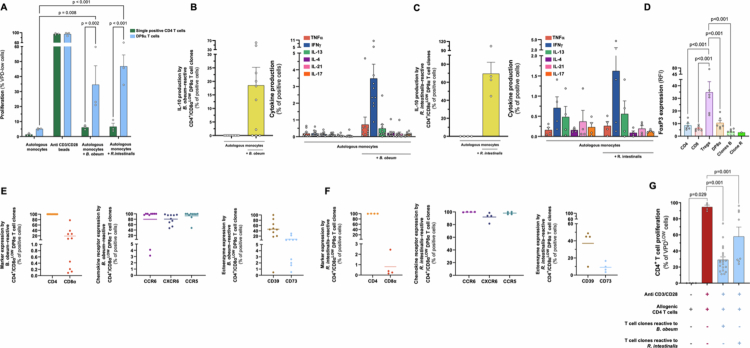
DP8α T cell responses to *Blautia obeum* and *Roseburia intestinalis.* (A). Purified CD4⁺ T cells (encompassing DP8α Tregs) from healthy donors' PBMCs were labeled with VPD and co-cultured with magnetically sorted autologous monocytes (1:1), loaded overnight or not with *B. obeum* DSM 25238 or *R. instestinalis* DSM 14610 (monocyte:bacteria ratio 1:5), in the presence of low-dose (20 IU/ml) rhIL-2. Five days later, T cell proliferation was assessed through VPD dilution. Proliferation in response to bacterial species are represented in percentages of VPD^LOW^ cells ± SEM. (B–G). Ten *B. obeum-* and 4 *R. intestinalis-*reactive DP8α T cell clones were obtained from healthy donors' PBMCs. Briefly, purified VPD-stained CD4^+^ T cells, comprising DP8α T cells, were co-cultured with purified autologous CD14^+^ monocytes loaded overnight with *B. obeum* DSM 25238 or *R. intestinalis* DSM 14610. Five days later, VPD^LOW^ CD3^+^/CD4^+^/CD8α^LOW^ cell clones were produced from 2 HDs using the FACS Aria III cell sorter. (B and C). The 10 *B. obeum-* and 4 *R. intestinalis-*reactive DP8α T cell clones were screened for their production of IL-10 as well as TNF-α, IFN-γ, IL-13, IL-4, IL-17 and IL-21 in response to autologous monocytes loaded ON with *B. obeum* DSM 25238 or *R. intestinalis* DSM 14610, respectively. IL-10 production is expressed in pg/mL ± SEM (background IL-10 production by bacteria-loaded monocytes cultured without T cell clones has been subtracted) and TNF-α, IFN-γ, IL-13, IL-4, IL-17, and IL-21 production are expressed as percentages of positive cells ± SEM, for the 10 *B. obeum*- (B) and the 4 *R. intestinalis*- (C) reactive DP8α T cell clones. FoxP3 expression has been assessed on the 10 *B. obeum-* and 4 *R. intestinalis-*reactive DP8α T cell clones and has been compared with the expression on polyclonal single positive CD4^+^ or CD8^+^ T cells, polyclonal DP8α T cells or CD4^+^/CD25^HIGH^/CD127^LOW^ polyclonal Tregs from 6 healthy donors. Data are represented as relative fluorescence intensity (RFI) ± SEM (D). (E and F). Expression of CD4, CD8α CCR6, CXCR6, CCR5, CD39, and CD73 were assessed by flow cytometry and data are represented as percentages of expression for the 10 *B. obeum-* (E) and the 4 *R. intestinalis-* (F) reactive DP8α T cell clones. (G). Allogeneic CD3/CD28-stimulated VPD-stained CD4^+^ cells from were co-cultured for 5 d with each *B. obeum-* and *R. intestinalis-*reactive DP8α T cell clones (ratio1:1). Proliferation was evaluated by VPD dilution. Data are represented as percentages of VPD^LOW^ cells ± SEM (G). Statistical significances were assessed using two-way ANOVA analyses (A), Wilcoxon U tests (B,C,G) or Kruskal–Wallis tests with Dunn's post hoc analyses (D). *p*-values < 0.05 were considered significant.

Our team has demonstrated that DP8α Tregs played a critical role in the protection against aGvHD^17^ and IBD.^9^ ^,^ ^11^ ^,^ ^26^ Several key commensal bacteria, particularly bacteria of *Blautia* and *Roseburia* genera, have also been associated with protection against aGvHD, gut diseases and other inflammatory ailments.^27-30^ *Blautia obeum* and *Roseburia intestinalis* represent key species of these genera. We therefore explored whether DP8α Tregs could also respond to these species. As above, VPD-labeled polyclonal CD4^+^ T cells derived from 3 HDs were co-cultured 5 d with autologous monocytes loaded with *B. obeum* DSM 25238 or *R. intestinalis* DSM 14610. At the end of the co-cultures, important fractions of DP8α T cells, but little to no SP CD4^+^ T cells, proliferated against both bacteria (Figure 5A). Indeed, *B. obeum*- and *R. intestinalis*-reactive cells represented over 34% and 46% of total DP8α T cells, respectively (Figure 5A). Ten *B. obeum*- and four *R. intestinalis*-reactive DP8α T cell clones were generated from these fractions (Figure S1). Upon activation, most *B. obeum*-reactive DP8α T cell clones secreted low to no IL-10, IFNγ and barely any of the other pro-inflammatory cytokines tested (Figure 5B). Activated *R. intestinalis*-reactive DP8α T cell clones all produced modest amounts of IL-10 (mean = 69.6 pg/mL ± 12.6) and none of the pro-inflammatory cytokines tested, with the exception of one clone that displayed IFNγ production at a low level, below 5% (Figure 5C). DP8α T cell clones of both specificities failed to express FoxP3 (Figure 5D). *B. obeum*-reactive DP8α T cell clones expressed CD4 and various percentages of clonal cells co-expressed CD8α, ranging from 0.1% to 77% (Figure 5E), but CD8α expression level was not associated with functional properties. All *B. obeum*-reactive DP8α T cell clones expressed particularly high levels of CCR6, CXCR6, and CCR5, except 2 clones expressing less than 5% of CCR6 (Figure 5E). While these clones expressed elevated levels of CD39 (46% ± 9.7% were CD39^+^), they expressed relatively low levels of CD73 (8.3% ± 3.1% (Figure 5E), as compared to both *F. duncaniae*- and *A. muciniphila*-reactive DP8α Treg clones (Figures S2F and 2I). Regarding *R. intestinalis*-reactive DP8α T cell clones, they all expressed CD4, but few cells co-expressed CD8α, below 3% (Figure 5F). Similar to *B. obeum*-reactive DP8α T cell clones, *R. intestinalis*-reactive DP8α T cell clones expressed high levels of CCR6, CXCR6, and CCR5 (above 81% of positive cells), as well as 37% ± 9.1% and 9.1% ± 2.8% of the cells expressed CD39 and CD73, respectively (Figure 5F). In terms of suppressive activity, *B. obeum*-reactive DP8α T cell clones inhibited 70% of CD4^+^ T cell proliferation as a mean (Figure 5G). Interestingly, 2 out of 4 *R. intestinalis*-reactive DP8α T cell clones did not inhibit CD4^+^ T cell proliferation from several HDs (Figure 5G). The other 2 clones exerted a significant suppression (mean = 70.5% of proliferation inhibited) (Figure 5G). Therefore, *B. obeum*- and *R. intestinalis*-reactive DP8α T cells can exert regulatory functions, however, *R. intestinalis*-reactive DP8α T cells appear less efficient/consistent in doing so.

### *Bacteria of Faecalibacterium*, *Akkermansia*, *Blautia,* and *Roseburia* genera effects on mo-DCs and their tolerogenic properties

**Figure 6.**
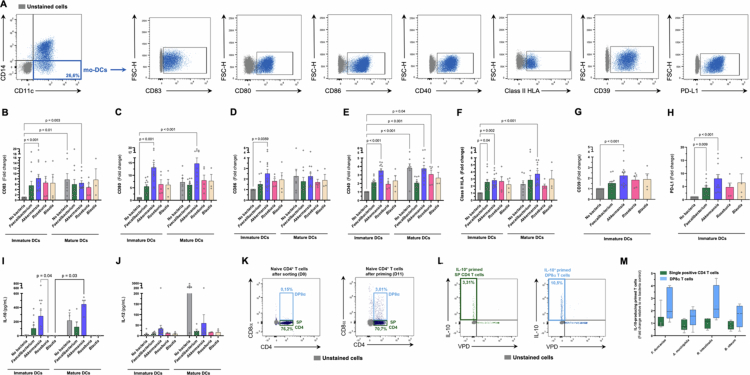
Bacteria of *F. duncaniae*, *A. muciniphila*, *B. obeum,* and *R. intestinalis* species effects on mo-DCs and their tolerogenic properties. Monocytes obtained through CD14-mediated magnetic sorting (Miltenyi) from healthy donors' PBMCs (*n* = 1–8), were differentiated into immature mo-DCs during a 5-d culture in the presence of 300 IU/ml rhIL-4 and 1000 IU/ml rhGM-CSF. mo-DCs were then incubated in the presence of R848 (a TLR7/8 agonist) (“mature DCs”) or not (“immature DCs”) for 48 h with different bacterial strains. Three strains of *F. duncaniae* (A2-165, FAM 10, and FAM 11), 3 strains of *A. muciniphila* (DSM 22959, FAM 49, and FAM 52), 3 strains of *R. intestinalis* (DSM 14610, FAM 252 or FAM 253), 1 strain of *B. obeum* (DSM 25238) and 1 strain of *B. luti* (FAME 138) have been tested (mo-DC:bacteria ratio 1:25). When the TLR agonist was used, DCs were incubated with bacteria overnight, followed by the addition of R848 to the co-culture for 48 h. In the absence of the agonist, DCs were exposed to bacterial strains for the entire duration of the co-culture without any additional agents. (A). The gating strategy used to study the phenotype of CD11c^+^/CD14^−^ mo-DCs is shown. (B–H). Expression levels of CD83 (B), CD80 (C), CD86 (D), CD40 (E), HLA-II (F), PD-L1 (G), and CD39 (H) were quantified by flow cytometry and represented as relative fluorescence intensity (RFI) ± SEM, normalized to the “No bacteria” control in immature DCs. I,J. IL-10 (I) and IL-12p70 (J) productions were assessed by ELISA and are represented in pg/mL ± SEM. (K–M). Sorted VPD-stained naive CD4^+^ T cells from healthy donors' PBMCs were co-cultured with allogeneic DCs previously exposed or not to *F. duncaniae* A2-165, *A. muciniphila* DSM 2295*9, B. obeum* DSM 25238 or *R. intestinalis* DSM 14610 overnight and matured with R848 for 48 h. After 11 d, CD4^+^ T cells were restimulated with CD3/CD28-coated beads. IL-10 secretion of single positive CD4^+^ and DP8α VPD^LOW^ primed T cells was assessed using the IL-10 secretion assay. A representative example is shown from one donor for the CD4/CD8α phenotype (K) and IL-10 production (L) of primed naïve CD4⁺ T cells from DCs exposed to *B. obeum*. Distributions, including median, interquartile range, minimum and maximum values, are shown for the all 6 donors tested (M). Statistical significances were assessed using Kruskal–Wallis tests with Dunn's post hoc analyses (B–J) or Mann–Whitney U tests (M). *p*-values < 0.05 were considered significant.

To investigate the bacterium-dependent underlying mechanisms leading to the priming/induction of specific DP8α Tregs, we assessed the effects of different bacterial strains belonging to the *Faecalibacterium*, *Akkermansia*, *Blautia,* and *Roseburia* genera on DC function. The gating strategy and examples of each staining are shown in Figure 6A. To this end, sorted monocytes were differentiated into immature mo-DCs, which were matured or not with R848, a potent pro-inflammatory TLR7/8 ligand. Both immature and matured mo-DCs were exposed to each bacterial strain for 48 h. Immature mo-DCs were used to examine the effects of bacterial strains on APCs in healthy homeostatic conditions. As expected, each of these bacterial strains, especially the one belonging to *Akkermansia* genera, matured mo-DCs as they induce CD83 expression on immature mo-DCs upon exposure (Figure 6B). As expected, R848-matured mo-DCs had a marked pro-inflammatory profile (Figures 6B–J and S3) allowing to clearly detect whether studied bacterial strains could efficiently counteract DC maturation. Bacterial strains of *Faecalibacterium*, *Akkermansia* and *Roseburia* genera tended to lessen CD83 expression on R848-matured mo-DCs (Figure 6B), as our team has previously shown with the *F. duncaniae* A2-165 strain on LPS-matured DCs.^7^ While *Faecalibacterium*, *Blautia, and Roseburia* strains limited maturation-induced expression of co-stimulation molecules, i.e., CD86 (Figure 6D) and CD40 (Figure 6E), *Akkermansia* strains failed to do so (Figure 6C–E). Class II HLA molecules were not significantly decreased by any of the bacterial strains tested of the 4 genera (Figure 6F). PD-L1 and CD39, two inhibitory receptors potentially promoting Treg induction, tended to be over-expressed by immature mo-DCs exposed to bacteria, particularly those belonging to the *Akkermansia* genera (*p* < 0.001) (Figure 6G and H). *Faecalibacterium* strains, as already shown by us and others,^6-8^ as well as *Akkermansia* strains, could induce immature mo-DCs to produce IL-10, the main cytokine driving type-1-like Tregs (Tr1-like) (Figure 6I). Tested bacterial strains of *Blautia* and *Roseburia* did not induce IL-10 production (Figure 6I). Remarkably, the 4 bacterial genera inhibited R848-matured DCs to secrete IL-12p70 (Figure 6J). In summary, while different bacterial strains belonging to all 4 genera licensed mo-DCs with tolerogenic features, the effects differed between genera.

Importantly, mo-DCs exposed to *F. duncaniae* A2-165, *A. muciniphila* DSM 22959, *B. obeum* DSM 25238 or *R. intestinalis* DSM 14610 primed IL-10-producing specific DP8α T cells, but not SP CD4^+^ T cells, suggesting that these bacteria could directly promote DCs to preferentially prime specific DP8α Tregs (Figure 6K–M).

## Discussion

Our team has initially identified that DP8α Tregs could recognize *F. duncaniae* A2-165 in a TCR-dependent manner.^9^ Here we showed that bacterial strains of *A. muciniphila* (Figures 2 and 4), *B. obeum* (Figure 5A and B), and *R. intestinalis* (Figure 5A and C) can also be recognized by DP8α Tregs. While some DP8α Treg clones kept producing residual cytokine in the presence of an HLA class II blocking antibody (Figure 2D), TCR-dependency of these clonal responses have nevertheless been established. Such a residual cytokine production could possibly be due to an incomplete blockade, triggering of potential TCR-independent pathways by bacteria and/or clone to clone variability in TCR affinities. The DP8α Treg subset therefore seems particularly prone to respond to commensal bacteria and likely represents key players in gut homeostasis maintenance and tolerance to gut microbiota. One possibility to explain the apparent specialization of DP8α Tregs to respond to gut commensal bacteria could rely on the fact that these CD4^+^ T cells be primed in one gut location, where specific factor(s) for such location would possibly, in parallel, induce CD8α expression. Such a hypothesis would be consistent with the presence of several other gut-resident CD8αα-positive cell subsets, e.g., innate-like natural CD8αα^+^ or CD4^+^/CD8αα^+^ intraepithelial lymphocytes,^42^ as well as CD8αα^+^ MAIT cells,^43^ likely through locally abundant cytokines such as TGFβ1^44^ and IL-15.^45^

DP8α Tregs are remarkably abundant in the colonic lamina propria,^9^ ^,^ ^11^ where they are in the vicinity of gut microbiota. DP8α Tregs could therefore likely be induced in the gut by commensal-exposed tolerogenic APCs, themselves also presenting bacterial antigens, which could explain their TCR specificities. It remains to be determined whether each commensal bacterium directly induces specific DP8α Tregs through its own effects on APCs, or whether a limited number of bacteria could tolerogenize APCs presenting T cell antigens/epitopes shared among a panel of bacteria, thereby enabling DP8α Tregs to recognize them *via* cross-reactivity. Each bacterial species could indeed theoretically induce actual tolerogenic DCs, likely through different mechanisms as our results showed that they differentially affect DC maturation, phenotype and their cytokine production (Figure 6B–J). Furthermore, DCs exposed to each bacterial species appear capable of priming naive CD4^+^ T cells into DP8α cells producing IL-10 (Figure 6K and L), supporting the above hypothesis. Additional experiments, involving the sorting of these IL-10-producing primed CD4^+^ T cells to assess their actual suppressive function, remain needed to unambiguously determine whether they are indeed DP8α Tregs. Another hypothesis would rely on the fact that one bacterium could induce the priming of DP8α Tregs through its effect on DCs, and that these primed DP8α Tregs could then be activated by other bacteria (without promoting tolerogenic DCs) upon encountering shared antigenic epitopes presented on class II HLA molecules with the original anti-inflammatory bacteria.

DP8α T cell clones produced in this study exhibited similar profiles, with slight differences depending on their TCR specificities. For instance, upon activation, while *F. duncaniae*- and *A. muciniphila*-reactive DP8α clones produced IL-10 and as well as TNFα, IFNγ and IL-13 (Figures S2A and B and 2A–D), *B. obeum*- and *R. intestinalis*-reactive DP8α clones produced low levels of IL-10 and IFNγ (Figure 5B and C). These results strengthen the hypothesis that each bacterial species could induce tolerogenic DCs through distinct pathways, leading to the priming of several sub-populations of DP8α Tregs. Slighter differences between DP8α clones obtained against *F. duncaniae* and *A. muciniphila* were also observed, For instance, in terms of TNFα production (Figures 1 and D, 4D and E). In this particular example, while the TCR of DP8α Treg clones could respond to two molecules derived from each bacterium, there could be slight differences in the amino-acid sequences of the epitopes, minor enough to be presented on class II molecules, but modifying/lowering the TCR affinity and the subsequent signal transduction strength and cytokine production^46^ when stimulated by the second bacterium.

It is worth noting that DP8α Tregs can sustain substantial suppressive activity (Figures 3 and 5) despite generally producing some pro-inflammatory cytokines. This is in accordance with Tr1 cells exerting suppressive functions while producing pro-inflammatory cytokines like IFNγ.^47^

The observed heterogeneity in the expression levels of studied markers (CD8α, CCR6, CXCR6, CCR5, CD39, CD73) by DP8α Treg clones was not associated to their level of suppressive function, suggesting that, if involved, none of these markers directly drive the suppressive function of these cells by itself.

Decrease in the abundance of the 4 bacterial species used in this study have been extensively reported to be associated with various pathological conditions, such as IBD, metabolic disorders, autoimmunity or detrimental allo-reactive responses in transplantation settings like GvHD.^16^ ^,^ ^18-20^ ^,^ ^22-25^ ^,^ ^48^ *A. muciniphila*, alike *F. duncaniae*, can promote anti-tumor immunity and significantly improve the clinical response to immune checkpoint blockade.^6^ ^,^ ^35-37^ ^,^ ^49^ Further studies are needed to uncover how DP8α Tregs, or the lack thereof, could be implicated in this myriad of conditions.

Our team has reported the substantial protective effect of DP8α Tregs in IBD^9^ ^,^ ^11^ and aGvHD.^17^ Alongside studies aiming at further associating diseases' development and progression with alterations in the abundance of specific gut bacteria, as well as further characterizing DP8α Tregs with regards to their antigen specificities should help pinpointing additional pathological contexts in which DP8α Tregs could represent a therapeutic candidate. Such strategies could rely on adoptive DP8α Treg transfer and/or administration of antigens, e.g., in the form of probiotics, to activate these Tregs *in vivo*.

**Table 1.** Description of the bacterial strains used in the study.

Designation	Phylum	Family	Strains	Source	Origin
*Faecalibacterium duncaniae*	Bacillota	Oscillospiraceae	A2-165	Human, female, 34 y, fecal sample	INRAE
*Faecalibacterium duncaniae*	Bacillota	Oscillospiraceae	FAM 10	Human feces	MaaT Pharma
*Faecalibacterium duncaniae*	Bacillota	Oscillospiraceae	FAM 11	Human feces	MaaT Pharma
*Akkermansia muciniphila*	Verrucomicrobiota	Akkermansiaceae	DSM 22959	Human intestinal tract (feces)	INRAE
*Akkermansia muciniphila*	Verrucomicrobiota	Akkermansiaceae	FAM 49	Human feces	MaaT Pharma
*Akkermansia muciniphila*	Verrucomicrobiota	Akkermansiaceae	FAM 52	Human feces	MaaT Pharma
*Akkermansia muciniphila*	Verrucomicrobiota	Akkermansiaceae	FAM 66	Human feces	MaaT Pharma
*Akkermansia muciniphila*	Verrucomicrobiota	Akkermansiaceae	FAM 272	Human feces	MaaT Pharma
*Akkermansia muciniphila*	Verrucomicrobiota	Akkermansiaceae	FAM 278	Human feces	MaaT Pharma
*Blautia obeum*	Bacillota	Lachnospiraceae	DSM 25238	Human feces	INRAE
*Blautia luti*	Bacillota	Lachnospiraceae	FAME 138	Human feces	MaaT Pharma
*Roseburia intestinalis*	Bacillota	Lachnospiraceae	DSM 14610	Infant feces	INRAE
*Roseburia intestinalis*	Bacillota	Lachnospiraceae	FAM 252	Human feces	MaaT Pharma
*Roseburia intestinalis*	Bacillota	Lachnospiraceae	FAM 253	Human feces	MaaT Pharma

## Supplementary Material

Supplementary material Supplementary Material

Supplementary figures.pdf Supplementary figures.pdf

## Data Availability

The data that support the findings of this study are openly available in Mendeley Data at http://doi.org/10.17632/v7sm5swy79.1 (DOI: 10.17632/v7sm5swy79.1).
