# Supplementary material for: Identification of human DP8α regulatory T cell sub-populations reactive to health-associated anti-inflammatory gut commensals
Source: Gut Microbes. 2026 Jun 28;18(1):2690686. doi: 10.1080/19490976.2026.2690686 (PMC13321884; doi:10.1080/19490976.2026.2690686)
Supplement: Supplementary material [file KGMI_A_2690686_SM2203.docx]

**Supplementary Figure 1. *F. duncaniae*–reactive DP8α Treg clones’ cytokine production and phenotype.**

Eight *F. duncaniae*-reactive DP8α Treg clones obtained from HD-derived PBMCs. Briefly, purified VPD-stained CD4^+^ T cells, comprising DP8α T cells, were co-cultured with purified autologous CD14^+^ monocytes loaded overnight with *F. duncaniae*. Five days later, VPD^LOW^ CD3^+^/CD4^+^/CD8α^LOW^ cell clones were produced from 2 HDs using the FACS Aria III cell sorter. **A,B.** All *F. duncaniae*-reactive DP8α T cell clones were screened for their production of IL-10 as well as TNF-α, IFN-γ, IL-13, IL-4, IL-17 and IL-21 in response to autologous monocytes loaded with *F. duncaniae.* IL-10 production is represented in pg/mL ± SEM (background IL-10 production by bacteria-loaded monocytes cultured without T-cell clones has been subtracted) (**A**) and TNF-α, IFN-γ, IL-13, IL-4, IL-17 and IL-21 production is represented in percentages of positive cells ± SEM (**B**). The expression of FoxP3 has been assessed on all *F. duncaniae* DP8α T cell clones and has been compared with the expression on polyclonal single positive CD4^+^ or CD8^+^ T cells, polyclonal DP8α T cells or CD4^+^/CD25^HIGH^/CD127^LOW^ polyclonal Tregs from 6 healthy donors. Data are represented as RFI ± SEM (**C**). **E,F.** The expression of CD4 and CD8α (**D**), CCR6, CXCR6 and CCR5 (**E**), as well as CD39 and CD73 (**F**) were assessed by flow cytometry and data are represented as percentages of expression. Statistical significances were assessed using Wilcoxon U tests (**A**) or Kruskal–Wallis tests with Dunn’s post hoc analyses (**C**). P-values <0.05 were considered significant.

**Supplementary Figure 2. Phenotype of immature versus R848‑matured monocyte‑derived dendritic cells**

Monocytes obtained through CD14-mediated magnetic sorting (Miltenyi) from healthy donors’ PBMCs (n = 1-8), were differentiated into immature mo-DCs during a 5-day culture in the presence of 300 IU/ml rhIL-4 and 1000 IU/ml rhGM-CSF. mo-DCs were then incubated in the presence of R848 (a TLR7/8 agonist) (“R848-matured DCs”) or not (“immature DCs”) for 48 h. Expression levels of CD83 (**A**), CD80 (**B**), CD86 (**C**), CD40 (**D**) and HLA-II (**E**) were quantified by flow cytometry and represented as relative fluorescence intensity (RFI) ± SEM. Statistical significances were assessed using Wilcoxon tests. P-values <0.05 were considered significant.
