# Supplementary material for: Identification of human DP8α regulatory T cell sub-populations reactive to health-associated anti-inflammatory gut commensals
Source: Gut Microbes. 2026 Jun 28;18(1):2690686. doi: 10.1080/19490976.2026.2690686 (PMC13321884; doi:10.1080/19490976.2026.2690686)
Supplement: Supplementary figures.pdf [file KGMI_A_2690686_SM1258.pdf]

A

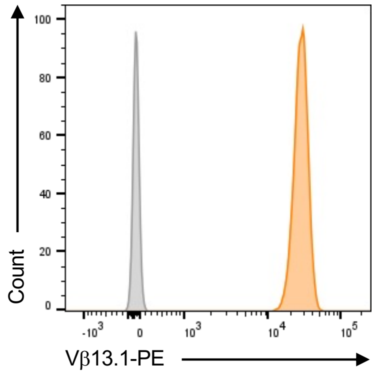

B

DP8α T cell clone reactive to ***A. muciniphila***	TCR-Vβ
Clone A1	Hu-Vβ 7.1
Clone A2	Hu-Vβ 9
Clone A3	Hu-Vβ 5.1
Clone A4	Hu-Vβ 8
Clone A5	Hu-Vβ 22
Clone A6	ND
Clone A7	Hu-Vβ 9
Clone A8	ND

DP8α T cell clone reactive to ***B. obeum***	TCR-Vβ
Clone B1	ND
Clone B2	Hu-Vβ 13.6
Clone B3	Hu-Vβ 13.1
Clone B4	Hu-Vβ 7.2
Clone B5	Hu-Vβ 3
Clone B6	Hu-Vβ 5.1
Clone B7	Hu-Vβ 5.1
Clone B8	ND
Clone B9	Hu-Vβ 2
Clone B10	ND

DP8α T cell clone reactive to ***R. intestinalis***	TCR-Vβ
Clone R1	ND
Clone R2	ND
Clone R3	Hu-Vβ 13.1
Clone R4	Hu-Vβ 9

Supplementary Figure 1. TCR Vβ chains expressed by DP8α clones

All generated DP8α clones were assessed for their TCR Vβ chain expression pattern through flow cytometry using the IOTest® Beta Mark TCR V beta Repertoire kit (Beckman Coulter). Most clones could be stained as 70 % of human Vβ chains can be detected. Cells stained with specific antibodies expressed a single Vβ chain, with > 99 % of positive cells, demonstrating cultures were clonal. A *B. obeum* DP8α clone (clone B3) is shown as a representative example (**A**) and Vβ chains from other clones are indicated in the tables (**B**).

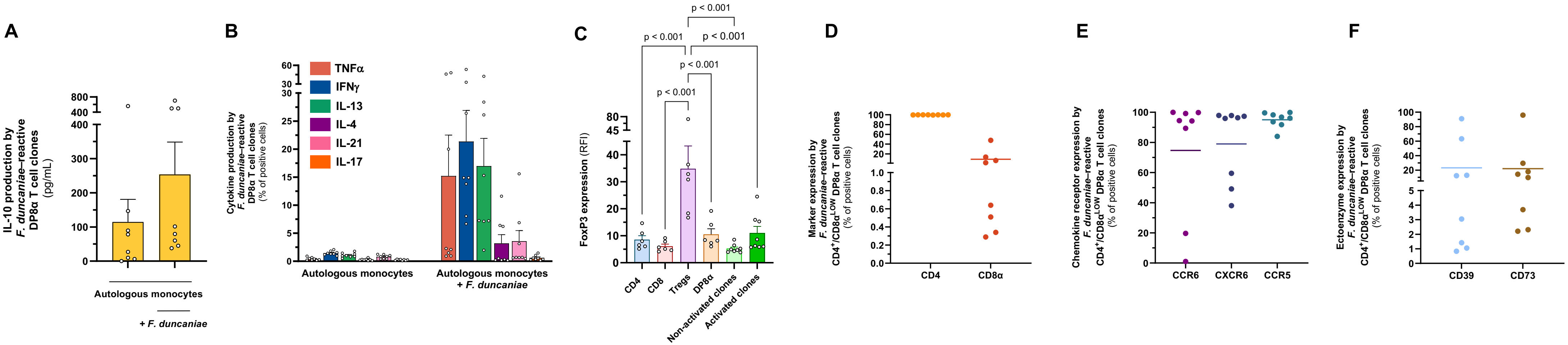

Supplementary Figure 2. *F. duncaniae*–reactive DP8α Treg clones' cytokine production and phenotype

Eight *F. duncaniae*-reactive DP8α Treg clones obtained from HD-derived PBMCs. Briefly, purified VPD-stained $CD4^+$ T cells, comprising DP8α T cells, were co-cultured with purified autologous $CD14^+$ monocytes loaded overnight with *F. duncaniae*. Five days later, VPD^{LOW} $CD3^+/CD4^+/CD8\alpha^{LOW}$ cell clones were produced from 2 HDs using the FACS Aria III cell sorter. **A,B.** All *F. duncaniae*-reactive DP8α T cell clones were screened for their production of IL-10 as well as TNF-α, IFN-γ, IL-13, IL-4, IL-17 and IL-21 in response to autologous monocytes loaded with *F. duncaniae.* IL-10 production is represented in pg/mL ± SEM (background IL-10 production by bacteria-loaded monocytes cultured without T-cell clones has been subtracted) (**A**) and TNF-α, IFN-γ, IL-13, IL-4, IL-17 and IL-21 production is represented in percentages of positive cells ± SEM (**B**). The expression of FoxP3 has been assessed on all *F. duncaniae* DP8α T cell clones and has been compared with the expression on polyclonal single positive $CD4^+$ or $CD8^+$ T cells, polyclonal DP8α T cells or $CD4^+/CD25^{HIGH}/CD127^{LOW}$ polyclonal Tregs from 6 healthy donors. Data are represented as RFI ± SEM (**C**). **E,F.** The expression of CD4 and CD8α (**D**), CCR6, CXCR6 and CCR5 (**E**), as well as CD39 and CD73 (**F**) were assessed by flow cytometry and data are represented as percentages of expression. Statistical significances were assessed using Wilcoxon U tests (**A**) or Kruskal–Wallis tests with Dunn's post hoc analyses (**C**). P-values <0.05 were considered significant.

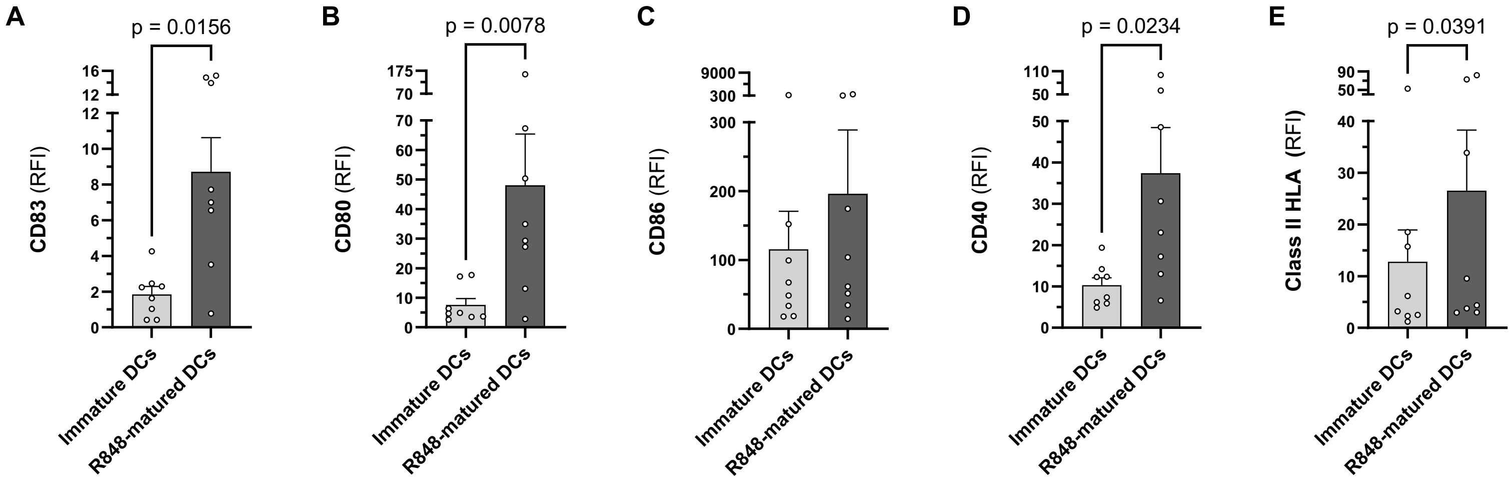

Supplementary Figure 3. Phenotype of immature versus R848-matured monocyte-derived dendritic cells

Monocytes obtained through CD14-mediated magnetic sorting (Miltenyi) from healthy donors' PBMCs (n = 1-8), were differentiated into immature mo-DCs during a 5-day culture in the presence of 300 IU/ml rhIL-4 and 1000 IU/ml rhGM-CSF. mo-DCs were then incubated in the presence of R848 (a TLR7/8 agonist) ("R848-matured DCs") or not ("immature DCs") for 48 h. Expression levels of CD83 (**A**), CD80 (**B**), CD86 (**C**), CD40 (**D**) and HLA-II (**E**) were quantified by flow cytometry and represented as relative fluorescence intensity (RFI) ± SEM. Statistical significances were assessed using Wilcoxon tests. P-values <0.05 were considered significant.
